# Association of G/C (rs638405) Polymorphism in *β-secretase* Gene with Alzheimer’s Disease

**Published:** 2018

**Authors:** Mostafa Chashmpoosh, Hossein Babaahmadi, Rouhollah Mousavidehmordi, Bita Shalbafan, Asma Mohammadi, Alireza Kheirollah

**Affiliations:** 1. Department of Biochemistry, Faculty of Medical, Cellular and Molecular Research Center, Ahvaz Jundishapur University of Medical Sciences, Ahvaz, Iran; 2. Department of Neurology, Faculty of Medical, Ahvaz Jundishapur University of Medical Sciences, Ahvaz, Iran

**Keywords:** Alzheimer’s disease, Amyloidogenic proteins, *BACE1* gene, Genotype, Iran

## Abstract

**Background:**

Alzheimer’s Disease (AD) is a neurodegenerative disorder, which is the most common cause of dementia in the elderly. Accumulation of β-amyloid plaques outside neurons is the most important pathological hallmark of AD, which is produced by cleavage of amyloid precursor protein by the Alzheimer’s β-secretase (*BACE1*). Since *BACE1* is a key enzyme in the formation of β-amyloid peptides, the purpose of this study was to assess the association between polymorphisms of G/C (rs638405) *BACE1* gene with sporadic AD in Khuzestan, Isfahan and Fars provinces in Iran.

**Methods:**

Genotypes were determined by the PCR–Restriction Fragment Length Polymorphism (PCR–RFLP) technique in two groups including 89 sporadic AD patients and 73 healthy subjects.

**Results:**

The findings of the *BACE1* G/C (rs638405) polymorphism revealed that there was no significant difference between AD patients and controls in men group; however, there was a weak difference in the frequency of CC genotype between patients and controls in women group (*χ*^2^=3.333, df=1, p=0.068).

**Conclusion:**

The results of this study suggest that the G/C (rs638405) polymorphism of *BACE1* gene might not be related with sporadic AD in Khuzestan, Isfahan and Fars provinces in Iran. However, our results do not support a genetic risk factor of this polymorphism for developing AD in male group of this study.

## Introduction

Alzheimer’s Disease (AD) is the most common cause of dementia that more than 15 million people are suffering from this disease worldwide. This disease is a neurodegenerative disorder and its most important pathological characteristics are β-amyloid plaques and neurofibrillary tangles that are formed through the accumulation of β-amyloid peptides outside neurons and hyperphosphorylated tau protein within neurons, respectively 1,2. β-amyloid peptides are in two forms, 40 or 42 amino acids 3,4. There is a strong association between familial AD and increasing production of 42-amino acid β-amyloid peptide 5,6. Increase in β-amyloid peptides occurs years before the occurrence of AD symptoms; therefore, the increase in β-amyloid peptides will trigger AD pathology 7,8. β-amyloid peptides are produced through endoproteolysis of the Amyloid Precursor Protein (APP) that is a large type-I trans-membrane protein 9,10. Amyloid precursor protein which exists in all cells is cleaved by three proteases including α-secretase, β-secretase, and γ-secretase.

APP is first cleaved by α- or β-secretase and then the membrane-bound remaining is further cleaved by γ-secretase. β-secretase is a protease that acts in place of amino acid, aspartic acid and produces β-amyloid peptide and the C99 fragment, the 99 amino acids from the C-terminal of the amyloid precursor protein. Next, γ-secretase cleaves amyloid precursor protein and produces β-amyloid peptides with different sizes. α-secretase with the effect on the amyloid precursor protein produces C83 fragment. Because the α-secretase is not able to produce β-amyloid peptides longer, thus does not produce beta-amyloid plaques. But β-secretase and γ-secretase are able to produce β-amyloid peptides. Therefore, APP cleavage by β-secretase and then by γ-secretase is the very well known cause of AD by production and accumulation of β-amyloid plaques in the brain.

In this pathway, β-secretase is a key enzyme and its activation or inactivation is essential for creation and the treatment of AD 7. β-secretase has two isoforms including β-Secretase Enzyme in AD 1 (*BACE1*) and β-Secretase Enzyme in AD 2 (*BACE2*). These two enzymes are similar; however, *BACE1* is more important and is a key enzyme in the production of β-amyloid peptides and formation of β-amyloid plaques. The *BACE1* is a membrane-bound enzyme from pepsin family and can cleave peptide bind of aspartic and glutamic acid of amyloid precursor protein 7. Several factors have been reported to affect the *BACE1* activity and gene expression; for instance, hypoxia 11, heat shock 12 and cytokines 13,14.

In addition, Single Nucleotide Polymorphism (SNP) affects *BACE1* activity and gene expression. The *BACE1* gene is located on chromosome 11 (11q23.3) and it seems that genetic variation in this gene can increase the risk of AD. SNP is very important for making phenotypes. Although there are 23 genetic locations on *BACE1* gene for making polymorphism, few of these sites can increase the risk of AD 15. Many studies have been done on this subject. For example, Todd *et al* conducted a study on northern Irish population and showed that 11 genetic locations in *BACE1* gene are not related with risk of AD 16. However, polymorphism of G/C (rs638405) within exon 5 of the *BACE1* gene is especially important. Several case-control studies stated that it is possible that G allele of the *BACE1* gene increases the risk of AD 17–20, but other studies showed it is not related to increased risk of AD 21–24. In addition, several meta-analyses have been conducted on this polymorphism. For example, a meta-analysis of 9 case-control studies shows that there is not a significant relationship between polymorphism in exon 5 of *BACE1* gene and risk of AD 25. However, a study that has been done by Jo *et al* revealed that there is a weak relationship between this polymorphism and risk of AD in Asian population 23.

In addition, in other meta-analysis Wang *et al* proved that GG genotype and G allele of polymorphism of G/C (rs638405) within exon 5 of the *BACE1* gene possibly increase the risk of AD 26. However, in another study, Yu *et al* stated that polymorphism of G/C (rs638405) of the *BACE1* gene might decrease the risk of AD in Asian and APOE4 positive patients 15. Therefore, polymorphism of G/C (rs638405) within exon 5 of the *BACE1* gene can be related with the increased risk of AD 20. Thus, due to the growing prevalence of AD and the important role of some single nucleotide polymorphisms in AD risk, this study was carried out to evaluate the association between polymorphism of *BACE1* gene, G/C (rs638405), and sporadic AD in population of Khuzestan, Isfahan and Fars provinces in Iran.

## Materials and Methods

### Study subjects

A total of AD patients and controls were recruited from outpatient clinics of several medical centers. Using statistical formulas, the sample size for this study was about 300 in each group, but due to the rare nature of Alzheimer’s samples, relevant information was collected from 6 August, 2013 to 26 November, 2013 from three provinces of Khuzestan, Isfahan and Fars. Therefore, the number of samples for comparing this ratio in each group was about 80 people. The subjects in this project consisted of 162 individuals living in Khuzestan, Isfahan and Fars provinces in Iran. The AD group consisted of 89 patients (mean age= 72.40 years, SD=10.251, range=51–94) and the second group consisted of 73 healthy subjects as the control group (mean age=70.78 years, SD=6.112, range=65–91).

For the diagnosis of AD patients, medical examination, DSM-IV criteria and neurological tests including Computed Tomography (CT) or brain magnetic resonance imaging (MRI) and cognitive function tests, including Mini-Mental State Examination (MMSE) were used by an expert neurologist. Each control subject was identified by medical interview, medical history and cognitive function tests, including mini-mental state examination (MMSE). The control subjects had a MMSE score of ≥26. Control group was selected from individuals monitored by the neurologist from several medical centers in Ahvaz city. The informed consent was taken from the control group and due to the patient’s condition in AD group and lack of optimal consciousness in advanced stages, their guardians submitted the consent form. The ethics committee of Ahvaz Jundishapur University of Medical Sciences approved the procedure.

### Isolation of DNA and genotyping

Genomic DNA was isolated from whole blood samples with a standard procedure by using the QIAamp blood kit (QIAGEN, Hilden, Germany). Blood samples (2.0 *ml*) were collected in blood tubes, where they were stored or transported. For DNA isolation, the blood was transferred to processing tubes (filled with cell lysis buffer), and the solution was mixed with lyse red and white blood cells. Cell nuclei were pelleted by centrifugation, washed, and resuspended in digestion buffer. Protein contaminants were removed by incubation with a protease. DNA was precipitated in iso-propanol, washed in 70% ethanol, dried, and resuspended in resuspension buffer.

### Genotyping of BACE1 gene G/C (rs638405) polymorphism

Genotypes were determined by the PCR-restriction fragment length polymorphism (PCR-RFLP) technique. For G/C SNP of *BACE1* gene, the forward primer was 5′-CTGATCTTATTGCTTGGTCCTTGG G-3′ and the reverse primer was 5′-CTTATGTTCCCA GGCTCTCCCTTG-3′ 20. PCR was performed using 1 *μg* of genomic DNA in 7 *μl* sterile D.W, 13 *μl* master mix PCR (Taq DNA Polymerase 2x Master Mix RED; Ampliqon, Denmark) and 0.2 *μM* of each primer 27. The PCR cycling conditions were 5 *min* at 95*°C* followed by 30 cycles of 30 *s* at 95*°C*, 30 *s* at 59*°C* and 45 *s* at 72*°C*, with a final step at 72*°C* for 5 *min* to allow for the complete extension of all PCR fragments. To determine the genotypes of G/C (rs638405) polymorphism of *BACE1* gene, PCR product was digested with the BclI restriction enzyme (Thermo Scientific or Fermentas, Germany) at 55*°C* for 2 *hr*. The productions of enzymatic digestion were investigated by 2% agarose gel stained with safe stain under ultraviolet light.

### Statistical analysis

The data were analyzed by using SPSS program for windows version 18.0. Allelic frequencies were estimated by the allele counting method. To compare genotypes and alleles frequencies between AD patients and controls Chi-Square (*χ*^*2*^) test was used. For obtaining OR, Chi-Square (*χ*^*2*^) test was applied. The statistical significance was supposed at p<0.05.

## Results

### Genotypes and alleles frequencies in BACE1 gene polymorphism of G/C (rs638405)

**Figure 1.**
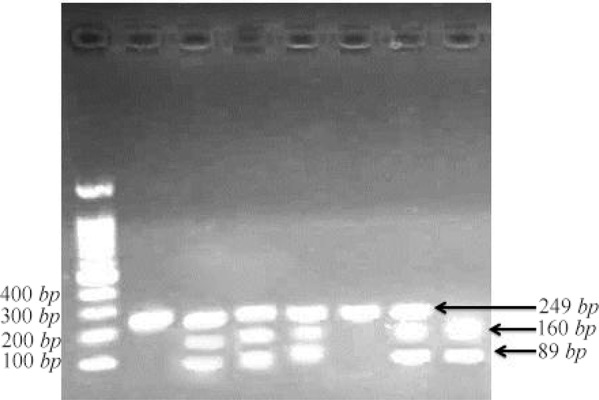
Digestion of PCR product with Bali.

The size of PCR products was 249 *bp*. In the case of *BACE1*, G allele generated two fragments of 160 and 89 *bp*, and in the case of C allele an uncut fragment of 249 *bp* was detected (Figure 1) 15. Next, the possible risk of AD associated with *BACE1* gene polymorphism of G/C (rs638405) was analyzed. For this aim, the demographic characteristics of the study subjects including 89 AD patients and 73 healthy controls were summarized. Frequency of men and women, the mean age and MMSE score for all subjects are shown in table 1. Chi-Square test and Mann-Whitney test were used to determine the relationship between sex and age with the AD, respectively. Unexpectedly, no statistically significant differences were found between sex and age with the AD and control populations (p>0.05). This means that sex and age may not be a confounding factor for AD. The results of allele and genotypes frequencies analysis in AD patients and control group are presented in table 2. Moreover, results of allele and genotypes frequencies analysis in AD patients and control group, when AD and control groups were stratified by sex, are shown completely in table 3. The findings of the study revealed that there was no significant difference in alleles and genotypes frequencies between AD patients and controls. However, when the AD patients and controls were stratified by sex, a weak difference was observed in the frequency of CC geno-type between AD patients and controls in women (*χ*^*2*^=3.333, df=1, p=0.068). In addition, there was a significant difference in C allele frequency between AD patients and controls in women (*χ*^*2*^=4.645, df=1, p=0.031) (Table 3).

**Table 1.** Frequency of sex distribution, the mean age, and MMSE score in AD and controls [polymorphism of G/C (rs638405) *BACE1* gene]

**Subjects**	**Number**	**Gender**		**Mean age**	**MMSE**

		**Male**	**Female**		
**AD patients**	89	34	55	72.40 years	Variable
**Controls**	73	32	41	70.78 years	26≤ MMSE
**Total**	162	66	96		

**Table 2.** The distribution of the *BACE1* genotypes and alleles frequencies in AD patients and control subjects

**Genotype/allele**	**Total**		**p-value**	**OR (95%CI)**

	**Patients**	**Controls**		
**GG**	4(2.5%)	2(1.2%)	0.414	Reference
**GC**	59(36.4%)	50(30.8%)	0.389	1.03(0.64–1.68)
**CC**	26(16.1%)	21(13.0%)	0.466	0.98(0.52–1.88)
**G**	67(37.6%)	54(37.0%)	0.237	Reference
**C**	111(62.4%)	92(63.0%)	0.182	1.01(0.67–1.53)

**Table 3.** The distribution of the BACE1 genotypes and alleles frequencies in AD patients and control subjects, stratified by sex in male and female subsets

**Genotype/allele**	**Male**		**p-value**	**OR(95%CI)**	**Female**		**p-value**	**OR(95%CI)**
	
	**Patients**	**Controls**			**Patients**	**Controls**		
**GG**	3(4.6%)	1(1.5%)	0.317	Reference	1(1.05%)	1(1.05%)	1.000	Reference
**GC**	25(37.5%)	20(30.3%)	0.456	0.58(0.4–1.81)	34(35.4%)	30.(31.2%)	0.617	1.18(0.63–2.23)
**CC**	6(9.1%)	11(16.6%)	0.225	1.95(0.66–5.72)	20(20.9%)	10(10.4%)	0.068	0.67(0.29–1.57)
**G**	31(45.6%)	22(34.4%)	0.216	Reference	36(32.7%)	32(39.0%)	0.628	Reference
**C**	37(54.4%)	42(65.6%)	0.574	1.21(0.63–2.31)	74(67.3%)	50(61.0%)	0.031	0.55(0.32–0.95)

## Discussion

AD is the most common cause of dementia and this disease is a neurodegenerative disorder. Its most important pathological characteristics are β-amyloid plaques and neurofibrillary tangles that are formed through the accumulation of β-amyloid peptides out-side neurons and hyperphosphorylated tau protein within neurons, respectively 1,2. Currently, examining the effective factors in the risk of developing AD can be useful for diagnosis and treatment of this problem 28. Researchers have shown that both genetic and environmental factors affect the risk of AD 29. Despite many studies that have been done in the field of diagnosis and treatment of AD, the cause of the disease has remained unknown 28. However, as mentioned before, genetic factors may affect the risk of developing AD.

One of these genetic factors is the SNP in genes related to proteins that are involved in the development of AD. For example, in one of our studies, it was found that polymorphism of G/A (rs34011) within *FGF1* gene and AA genotype and A allele may be associated with the risk of developing AD in people of Khuzestan, Isfahan and Fars provinces in Iran 30. Like the polymorphisms related to *APOE*, *CYP46A1* and *BDNF* genes have been studied in different countries and the results indicated that these polymorphisms can be associated with the risk of developing AD 31–33. In addition, the SNP related to *BACE1* gene can be considered as an important genetic risk factor for developing AD 23,34.

The *BACE1* is expressed in human pancreatic tissue and neurons in the brain at high levels 7. However, the pancreatic *BACE1* is in an inactive isoform and does not produce β-amyloid plaques 35,36. The *BACE1* is a key enzyme in the production of β-amyloid peptides; therefore, activation and inactivation of the *BACE1* is necessary for generation and remedy of β-amyloid plaques and any changes in this enzyme can be related to AD. The SNP affects *BACE1* gene activity and expression and can be related to AD. The *BACE1* gene is placed on chromosome 11 (11q23.3) 37. Several studies have been done on *BACE1* gene polymorphisms in AD patients, suggesting that G/C (rs638405) polymorphism within exon 5 of the *BACE1* gene is an important risk factor for causing and developing AD. For example, in one study, Kan *et al* reported that G/C (rs638405) polymorphism of *BACE1* gene and GG genotype are important genetic risk factors for developing AD in China 20. In another study, Gold *et al* evaluated polymorphism of G/C (rs638405) *BACE1* gene in association with APOε4 allele and indicated a synergetic effect between the G-allele and APOε4 allele on the risk of developing AD in Switzerland 37. However, Shi *et al* revealed that the C-allele was the risk factor for developing AD 38. However, Liu *et al* stated that G/C (rs638405) polymorphism of *BACE1* gene is not related with increased levels of β-amyloid plaques in brain tissue 22. The distributions of alleles or genotype frequencies of G/C (rs638405) in different populations suggest the ethnical variability in the populations.

In this study, G/C (rs638405) polymorphism of *BACE1* gene was analyzed for the first time in Khuzestan, Isfahan and Fars provinces of Iran. In agreement with other studies 22, our data showed that there was no significant difference in alleles and genotype frequencies between AD patients and controls. But our results, unlike a study that Nowotny *et al* conducted, evaluated polymorphism of G/C (rs638405) *BACE1* gene in association with APOε4 allele and they concluded that there was no significant difference in alleles and genotypes frequencies between AD patients and controls 19. In addition, Clarimón *et al* revealed a relationship between GG genotype of G/C (rs638405) gene in relation with APOε4 allele and AD 18, though our data stated that the G/C (rs638405) polymorphism of *BACE1* gene might not be related with sporadic AD in Khuzestan, Isfahan and Fars provinces in Iran. However, when the AD patients and controls were stratified by sex, our results revealed that there is a weak difference in the frequency of CC genotype between patients and controls in women. Also, Shi *et al* in agreement with our study stated that CC genotype of polymorphism of *BACE1* gene is associated with sporadic AD in Chinese Hans 38, although Kan *et al* indicated that there was a synergetic association between G-allele of G/C (rs638405) polymorphism and apolipoprotein E allele 4 with risk of Late-Onset AD in Chinese 20. A possible and more likely reason may be that the smaller number of male subsets than female subsets in AD patients is responsible for the lack of association between GG-homozygotes genotypes and G allele status with increases in the risk of AD in male subsets. Increasing the sample size to make sure whether the G/C (rs638405) polymorphism of *BACE1* gene is associated with the AD risk in Khuzestan, Isfahan and Fars provinces in Iran seems to be a necessary strategy to apply.

## Conclusion

In conclusion, the results of this study suggest that the G/C (rs638405) polymorphism of *BACE1* gene might not be related with sporadic AD in Khuzestan, Isfahan and Fars provinces in Iran, and the *BACE1* gene CC genotype frequency or C allele frequency might be a genetic risk factor for developing AD in Khuzestan, Isfahan and Fars provinces in Iran. Further studies in other populations with different ethnicities are needed to clarify the relationship of these polymorphisms with gene expression in AD patients. Polymorphism G/C (rs638405) in the *BACE1* gene is not the only polymorphism in this gene; other polymorphisms in *BACE1* gene may also increase the risk for AD. Synergistic interaction may exist between the polymorphism of the *BACE1* gene and other genes involved in the AD, such as the *ApoE* and *CYP46A1* genes 27. Therefore, further studies investigating the effects of the *BACE1* gene polymorphism are needed in the future to confirm the substantial role of this gene in the pathogenesis of AD.
